# Combined lipidomic and proteomic analysis of isolated human islets exposed to palmitate reveals time-dependent changes in insulin secretion and lipid metabolism

**DOI:** 10.1371/journal.pone.0176391

**Published:** 2017-04-27

**Authors:** Kirsten Roomp, Hjalti Kristinsson, Domitille Schvartz, Kumari Ubhayasekera, Ernest Sargsyan, Levon Manukyan, Azazul Chowdhury, Hannes Manell, Venkata Satagopam, Karlfried Groebe, Reinhard Schneider, Jonas Bergquist, Jean-Charles Sanchez, Peter Bergsten

**Affiliations:** 1 Luxembourg Centre for Systems Biomedicine, University of Luxembourg, Esch-Belval, Luxembourg; 2 Department of Medical Cell Biology, Uppsala University, Uppsala, Sweden; 3 Human Protein Sciences Department, Centre Médical Universitaire, University of Geneva, Geneva, Switzerland; 4 Analytical Chemistry, Department of Chemistry and Science for Life Laboratory, Uppsala University, Uppsala, Sweden; 5 Pivot Biomed Science GmbH, Trier, Germany; University of Rochester, UNITED STATES

## Abstract

Studies on the pathophysiology of type 2 diabetes mellitus (T2DM) have linked the accumulation of lipid metabolites to the development of beta-cell dysfunction and impaired insulin secretion. In most *in vitro* models of T2DM, rodent islets or beta-cell lines are used and typically focus is on specific cellular pathways or organs. Our aim was to, firstly, develop a combined lipidomics and proteomics approach for lipotoxicity in isolated human islets and, secondly, investigate if the approach could delineate novel and/ or confirm reported mechanisms of lipotoxicity. To this end isolated human pancreatic islets, exposed to chronically elevated palmitate concentrations for 0, 2 and 7 days, were functionally characterized and their levels of multiple targeted lipid and untargeted protein species determined. Glucose-stimulated insulin secretion from the islets increased on day 2 and decreased on day 7. At day 7 islet insulin content decreased and the proinsulin to insulin content ratio doubled. Amounts of cholesterol, stearic acid, C16 dihydroceramide and C24:1 sphingomyelin, obtained from the lipidomic screen, increased time-dependently in the palmitate-exposed islets. The proteomic screen identified matching changes in proteins involved in lipid biosynthesis indicating up-regulated cholesterol and lipid biosynthesis in the islets. Furthermore, proteins associated with immature secretory granules were decreased when palmitate exposure time was increased despite their high affinity for cholesterol. Proteins associated with mature secretory granules remained unchanged. Pathway analysis based on the protein and lipid expression profiles implicated autocrine effects of insulin in lipotoxicity. Taken together the study demonstrates that combining different omics approaches has potential in mapping of multiple simultaneous cellular events. However, it also shows that challenges exist for effectively combining lipidomics and proteomics in primary cells. Our findings provide insight into how saturated fatty acids contribute to islet cell dysfunction by affecting the granule maturation process and confirmation in human islets of some previous findings from rodent islet and cell-line studies.

## Introduction

Type 2 diabetes mellitus (T2DM) and obesity are closely linked [1, 2]. In obesity the pancreatic islet beta-cells respond to increased nutrient status and insulin resistance with increased insulin secretion [3]. Eventually the beta-cells in some individuals cannot maintain the required insulin secretion and glycemic control is lost. In our studies on isolated human islets mimicking the overfeeding state *in vitro* with elevated fatty acid levels (mainly palmitate) we have observed hypersecretion of insulin initially, followed by increased apoptosis and worsening of insulin secretion [4, 5]. This may translate to the *in vivo* setting, where fatty acids have opposite short- and long-term effects on insulin secretion [5–7]. Therefore, numerous studies have attempted to explore the molecular mechanisms behind the fatty-acid induced worsening of beta-cell function, often termed lipotoxicity [8]. To date the mechanisms put forth as driving forces of increased beta-cell apoptosis and reduced insulin secretion in response to palmitate include endoplasmic reticulum stress [9], mitochondrial dysfunction [10], formation of toxic ceramide species [11], altered lipid metabolism [12] and fatty acid binding G-protein coupled receptor signaling [4]. In one study the expression of exocytotic genes was shown to be decreased in T2DM patients [13]. Even though the involvement of these mechanisms have been substantiated separately, it is still not clear how the different mechanisms relate and depend on each other and what the driving phenomenon behind decreased insulin secretion in beta-cells exposed to elevated levels of saturated fatty acids is. To address this aspect another approach, which gives a broader perspective of the different events taking place, is needed. There are challenges when combining lipid- and proteomics data. Nevertheless such an approach or model is desirable as it could provide a deeper biological understanding of events taking place compared to the stand-alone lipid- or proteomic analysis. Also, most previous studies addressing lipotoxicity have been performed on rodent islets or cell-lines and to what extent the described mechanisms are valid also for human islets need to be verified since human and rodent islets characteristics differ in many aspects [14].

Therefore, our aim was to develop a method to investigate lipotoxicity in human islets by combining targeted lipidomics and un-targeted proteomics. We came up with a design where quantitative time-course lipidomics and proteomics were performed on human islets exposed to palmitate for different time periods with the aim to correlate the omics findings with the time-dependent effects of palmitate on insulin secretory response to glucose and insulin cellular content [4, 5].

## Materials and methods

### Human pancreatic islets

Human islets were obtained from the Islet Prodo Lab Inc. (Irvine, CA, USA). Ethical permission for the use and for the procedures and protocols involved in handling of human islets isolated from healthy individuals was obtained from the Regional Ethical Review Board in Uppsala, Sweden (EPN number 2010/006; 2010-02-10). All procedures involving islets were carried out in accordance with the aforementioned approval as well as in compliance with respective local guidelines and regulations. Islets were obtained from brain-dead otherwise healthy individuals from the Islet Prodo Lab Inc. (Irvine, CA, USA), where written informed consent of all donors was given prior by individual donors or their family members and documented. The obtaining and documenting of written consent for islet donation for research was done in line with the approved procedure by the Regional Ethical Review Board in Uppsala, Sweden (EPN number 2010/006; 2010-02-10) and according to respective local regulations and guidelines.

In total, islets from 6 donors were used: Donor 12133 (male, Asian, 62 years, BMI 26), Donor 12156 (female, Asian, 54 years, BMI 24.7), Donor 12240 (male, African American, 48 years, BMI 27.5), Donor 13017 (female, White, 47 years, BMI 20.6), Donor 13041 (female, African American, 59 years, BMI 25.7), Donor 13064 (male, White, 64 years, BMI 26.5). Purity of islets varied between 85–90%. As part of quality control, islets from each donor were analyzed to check for amounts of alpha- and beta-cells and fibroblasts via immunohistochemistry. Islets for insulin secretion measurements were picked individually to avoid non-islet structures. Human islets were cultured in CMRL 1066 medium containing 5.5 mM glucose and supplemented with 10% FBS and 0.5% fatty acid free bovine serum albumin (BSA) (fraction V; Roche Diagnostics GmbH, Mannheim, Germany), which was defined as control conditions. Experiments were started 4–7 days after the islet isolation.

### Treatment of islets with palmitate

Palmitate (Sigma Aldrich, St. Louis, MO, USA) was dissolved in 50% ethanol to a concentration of 100 mM. This stock solution was diluted in culture medium to 0.5 mM concentration and then allowed to complex for 30 min at 37°C with fatty acid free BSA to a final molar ratio of 6 to 1 as previously described [15]. Human islets were exposed to 0.5 mM palmitate for 0, 2 and 7 days, denoted “C”, “P2” and “P7”, respectively, as previously described [4]. All conditions received the same low concentration of ethanol in the buffer. During development of the model apoptosis marker cleaved caspase 3 (antibody; Cell signaling, USA) was measured routinely by western blotting on separate donors. Apoptosis was only significantly higher in palmitate-cultured islets compared to control conditions after 5 days of palmitate treatment. Control islets were cultured for 7 days in control conditions, P7 islets were cultured for 7 days in 0.5 mM palmitate and P2 islets were cultured for 5 days in control conditions and the final 2 days in 0.5 mM palmitate. Thus, all islets were cultured for 7 days. All secretion experiments were performed on the same day for all conditions for each donor.

### Insulin secretion

Groups of human islets (~15 islets) were collected and placed into a perifusion chamber as previously described [16]. In brief, islets were initially perifused for 1 hour at 37°C with a buffer containing 2  mM glucose, 125  mM NaCl, 5.9 mM KCl, 1.2  mM MgCl_2_, 1.3  mM CaCl_2_ and 25 mM HEPES, titrated to pH 7.4 with NaOH, and supplemented with 0.1% (w/v) fatty-acid free BSA. This was followed by 20 min perifusion with the same buffer containing 20 mM glucose. The perifusion rate was 170 μl/min. Perifusates were collected at -15, -10, -5, 0, 2, 4, 6, 10, 15, 20 minutes and amounts of secreted insulin measured. At 0 min the glucose concentration was raised from 2 to 20 mM. After perifusion, islets were washed with PBS and lysed in PBS containing 1% Triton X100 and 0.4% protease inhibitor cocktail (both obtained from Sigma Aldrich). Lysates were used for measurements of protein content. Insulin concentrations of secretion and cellular lysates were determined by ELISA (Mercodia AB, Uppsala, Sweden).

#### Proinsulin secretion

The experiments on proinsulin secretion were performed on islets obtained from four separate human donors. The same culture media and palmitate treatment was used as the first six donors. Two of the donor islets were supplied by Prodo (male, 43 years and female, 59 years) and two came from Uppsala University Islet Transplantation Unit (male, 78 years and unknown gender, 67 years). Secreted proinsulin and cellular proinsulin content were determined by ELISA (Mercodia AB).

#### Preparation and mass spectrometric analysis of proteins

Two hundred islets were lyophilized and resuspended in 100 μL 0.1% RapiGest—TEAB. After a brief sonication, six-plex TMT labeling was performed for protein quantification, according to manufacturer’s instruction (Thermo Scientific, Waltham, MA, USA). Briefly, 25 μg of each of the 6 samples were digested with trypsin, labeled, and pooled together, according to standard procedures [17]. Peptides were separated by off-gel electrophoresis, desalted and solubilized in an appropriate amount of 5% ACN / 0.1% formic acid for mass spectrometry analysis.

Nano LC-MS/MS analyses were performed on a nanoAcquity system (Waters, Milford, MA, USA) coupled to a LTQ-Orbitrap Velos Pro mass spectrometer (Thermo Scientific) as described elsewhere [17].

### Protein identification, quantification

Raw data were converted to mgf files, and peak lists were submitted to EasyProt [18] for research against the human UniProt/SwissProt database (20193 entries). Selected criteria for identification were <1% False Discovery Rate (FDR) and a minimum of 2 unique peptides per protein. Isobar [19] was used for quantification after isotopic correction, according to manufacturer instructions (Thermo Scientific).

Slight differences in the total channel intensity were typically observed in each of the channels, as the total amount of protein used for each TMT condition is not exactly the same. Therefore, the total channel intensities relative to each other were normalized; each peptide’s ion intensity was divided by a channel-specific factor derived from the comparison of the total sum of the ion counts, i.e. the normalization is made to assess an equal median intensity in each channel and the intensity of each peptide in that channel is then divided by one and the same factor. Next, the calculation of normalized quantifications was performed at the protein level, where each donor was treated separately. We applied the Libra methodology from the Trans-Proteomic Pipeline [20]: only proteins with at least two unique peptides were included. Each peptide channel was normalized by the sum of all channels of the respective peptide. The mean and population standard deviation for all peptides of a protein in each channel were determined. Subsequently, outlier removal was performed for peptides with intensities two standard deviations outside of the mean in one or more channels. The mean was then recalculated. If a peptide was measured twice due to different retention times and one displayed outlier characteristics, only the outlier peptide was removed. Contaminating proteins were excluded, as defined by Mascot [21]. This produced normalized intensities at the protein level.

### Donor-by-donor pathway analysis for proteomics

All proteins identified in each of the 6 donors were used for a donor-by-donor analysis. The protein lists for each donor and expression levels at the different time points were imported into Elsevier’s Pathway Studio (http://www.elsevier.com/online-tools/pathway-studio). This software was used to identify expert curated pathways. Each donor was examined separately and the pathways enriched in detected proteins were ranked; all pathways returned shared at least one member of the selection. Found pathways were sorted by the degree of similarity to the query collection, with the similarity score (p-value) being calculated as a ratio of a number of common objects between two pathways to the total number of objects in them.

Using the resulting six files of ranked pathways (one for each donor), a Ranking Enriched Pathways (*REP*) score was calculated for each pathway, as well as random ranks for that particular pathway as described previously [22]. This approach allows for direct comparisons of scores produced when pathways are ranked for the donors non-randomly and randomly. The score was calculated for each pathway: REP = rank_d1 * rank_d2 * rank_d3 * rank_d4 * rank_d5 * rank_d6/N^5, where *N* is the average of total number of pathways in all donors, and *rank_d1* to *rank_d6* are the ranks of particular pathways in each donor. Subsequently, the negative log of *REP* was taken in order to generate a positive value to enable sorting.

In order to visualize the six donors simultaneously, Cytoscape (http://www.cytoscape.org) was used as this was not possible in Pathway Studio. The interactions from Pathway Studio were exported to generate SIF files (Cytoscape Simple Interaction Format), the expression levels for each protein/functional class were exported to generate NOA files (Cytoscape Node Attribute File), both of which were then imported into Cytoscape to create the relevant nodes, relations and node attributes thus allowing the visualization of the top ranking pathway. Initially, two networks were generated, C vs. P2 and C vs. P7, each having the same layout for comparison purposes. Subsequently, P2 and P7 were compared in one network: the nodes are colored with the average expression level change for all donors for whom expression levels for that particular node existed.

### Time-point comparisons for proteomics

Comparisons of three time-points were made using the parametric, matched-pair t-test for the proteins found in all six donors (C vs. P2, C vs. P7 and P2 vs. P7). P-values were adjusted for multiple comparisons using the False Discovery Rate (FDR) method [23], using an adjusted p-value (q-value) cutoff of less than 0.15. Finally, proteins retained were those with fold changes less than 0.8 or greater than 1.2.

### Preparation and mass spectrometric analysis of lipids

One hundred islets were received from each treatment and washed 10 times with PBS. The sample was re-suspended in 400 μL of PBS and homogenized (Sonics Vibra Cell, Chemical instrument AB, Sweden). Whereas 350 μL of the sample was used for lipid extraction by employing different extraction techniques, 50 μL was set aside for total protein determination.

Aliquots of 150 μL, 150 μL and 50 μL were used for the lipid extraction for sphingolipids analysis [11], for free fatty acid analysis [24], and for cholesterol and oxycholesterols analysis [25], respectively. All the analyses were performed with LC-MS/MS (ABI 3200 Q trap linear mass spectrometer, Applied Biosystems, CA, USA) or GC-MS/MS (Scion TQ-GC-MS/MS system chromatograph, Bruker Daltonics Inc, Billerica, MA, USA) as previously described [24].

The lipids which were examined were the C14, C18:1, C16, C16:1, C20:1, C22, C22:1, C24, C24:1, C26 and C26:1 ceramides, the C14:0, C16:0, C16:1, C18:0, C18:1, C20:0, C20:1, C24:0 and C26:0 dihydroceramides, the C18:0, C18:1, C24:0, C24:1 and C26:0 glucosylceramides, the C16:1, C20:1, C22:1, C18, C16:0, C14:0, C20:0, C22:0, C24:1, C24:0, C26:0 and C26:1 sphingomyelins, dihydrosphingosine, sphingosine, palmitic acid, stearic acid, myristic acid, palmitoleic acid, oleic acid, linoleic acid, alpha-linolenic acid and cholesterol.

### Lipidomics normalization, statistical analysis

Lipid analytes were normalized to protein content by an in-house validated method (Dot-it-Spot-it protein assay, http://dot-it-spot-it.com/, Maple Stone AB, Uppsala, Sweden). Lipids were normalized to protein content to be consistent with the insulin cellular content analysis in this study, which is also normalized to protein content. Protein precipitate was homogenized and clarified and assessed according to the manufacturer’s instructions. The blackness intensity was quantified in each dedicated spot on the image with Image J (http://rsbweb.nih.gov/ij/). Protein concentrations were estimated by comparing the sample results with the results from a calibration curve using Rodbard curve fitting in Image J. Time-point comparisons were made for all three time-points with all six donors using the matched-pair t-test. P-values were adjusted using FDR, and a q-value cutoff of less than 0.05 was used.

### Integrated lipidomics and proteomics pathways

Information from databases especially relevant to lipids (LIPID MAPS [26], PubChem [27], KEGG [28]) was manually curated and integrated, due to a lack of resources with detailed information on the lipids we experimentally analyzed. Furthermore, enzymes which are active in these lipid pathways were included in the analysis. In the case of the fatty acid biosynthesis pathway, some branches of reactions, where neither proteins nor lipids were detected or measured, were excluded. Detailed pathways for fatty acid biosynthesis and sphingolipid metabolism were thus created.

## Results

### Insulin secretion

**Fig 1:**
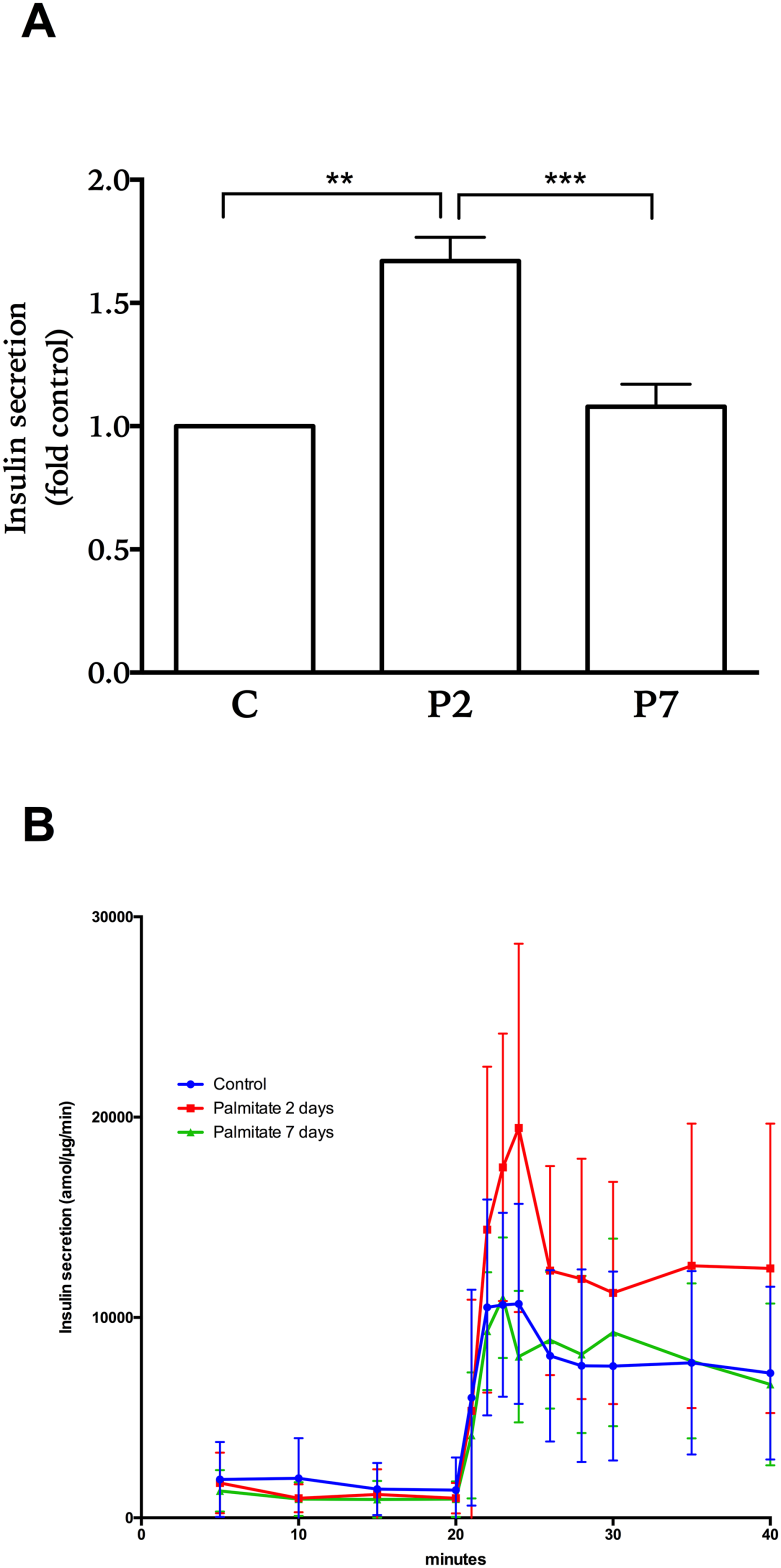
Glucose-stimulated insulin secretion from human islets treated with palmitate. Islets were treated with palmitate for two (P2) and seven (P7) days or without it in control conditions (C). Subsequently post culture, insulin secretion during glucose stimulation was assessed by perifusing islets with a buffer containing 2 mM glucose (first 20 minutes) followed by 20 mM glucose (last 20 minutes). Total amount of insulin secreted during glucose stimulation (A) and the kinetic secretory responses (B) are shown. Secretion amount from 6 donors was adjusted to total islet protein amount. Results show means ± SEM as fold control in panel A and ± SD in panel B. ** P<0.005 and *** P<0.0005 vs control.

Human islets treated for 2 days with palmitate secreted almost two times more insulin, when stimulated with 20 mM glucose, than islets treated in control conditions (Fig 1 After 7 days culture in the presence of palmitate insulin secretion was statistically significantly decreased compared with day 2, and was on average similar to levels of secretion observed from control islets (Fig 1).

### Insulin and proinsulin cellular content

**Fig 2:**
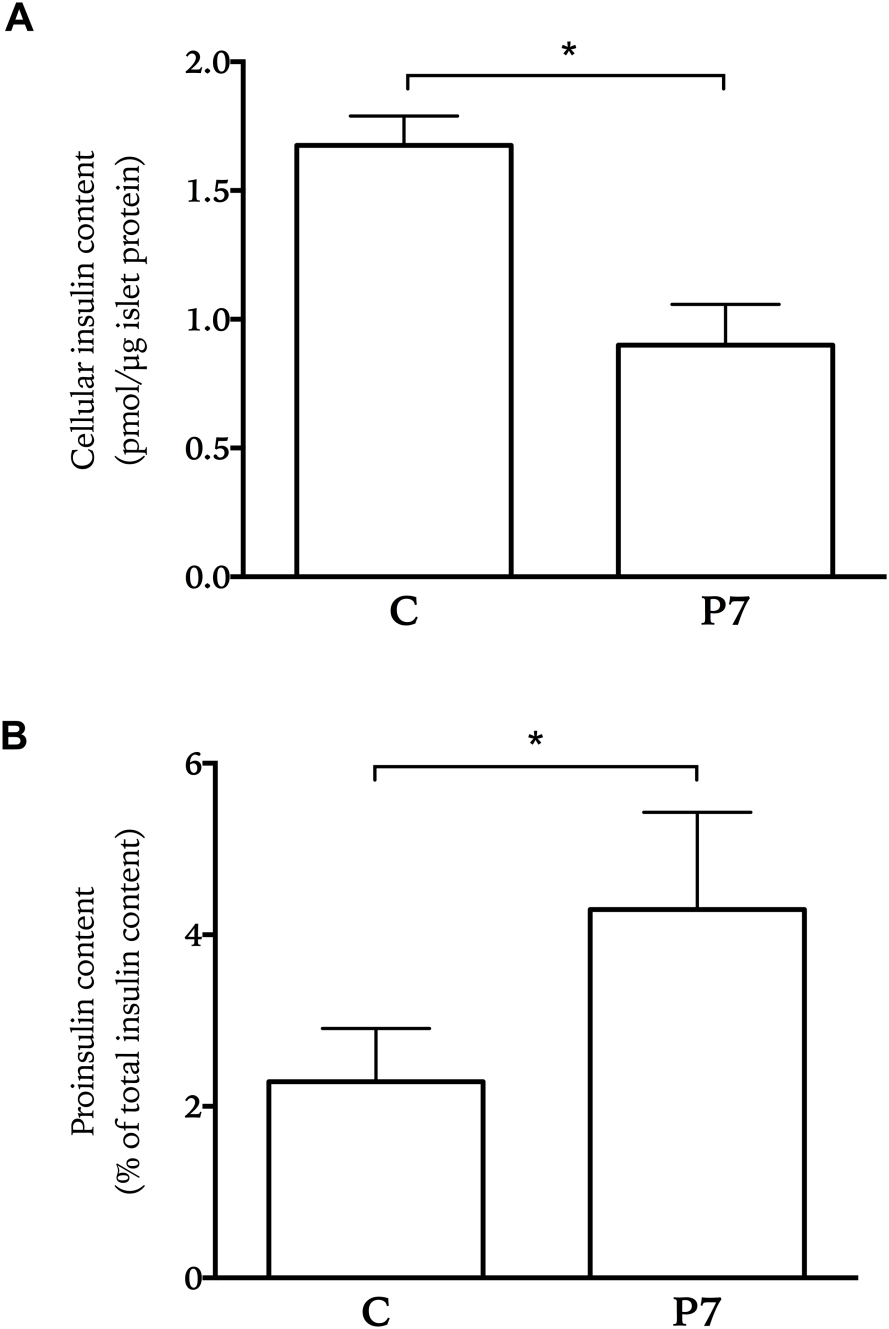
Insulin and proinsulin content in human islets treated with palmitate. Islets were treated for seven days with palmitate (P7). Subsequently, insulin and proinsulin content was measured. Results show means ± SEM as fold control (fold change of control) from 4 donors. * P<0.05 vs control.

To assess the effect of chronic palmitate culture on cellular insulin and proinsulin content, islets were treated for 7 days with palmitate. Cellular insulin content was significantly lower (about 50%) in islets treated for 7 days with palmitate than in control islets (Fig 2). Relative proinsulin cellular content (percentage of insulin content) doubled from 2% in control islets to 4% in islets treated for 7 days with palmitate (Fig 2).

### Time-point comparisons for proteomics: Differentially expressed proteins

Comparisons of the three time-points (C vs. P2, C vs. P7 and P2 vs. P7) identified 846 proteins found in all six donors at all time-points. Whereas no significantly differentially expressed proteins were identified in the control (C) vs. palmitate culture for 2 days (P2) comparison, 41 proteins were identified in the C vs. P7 comparison (Table 1), and 9 proteins were identified in the P2 vs. P7 comparison (8/9 were found in the C vs. P7 comparison). The single protein unique to the P2 vs. P7 comparison was COTL1, coactosin-like protein (SwissProt Accession Number Q14019).

**Table 1:** Differentially expressed islet proteins after palmitate treatment for 7 days.

	Protein Acc No	Gene Name	Description	Fold Change
1	P53396	ACLY	ATP-citrate synthase	1.32
2	P33121	ACSL1	Long-chain-fatty-acid—CoA ligase 1	1.23
3	P04075	ALDOA	Fructose-bisphosphate aldolase A	1.25
4	P63010	AP2B1	AP-2 complex subunit beta-1	1.51
5	Q13838	BAT1	Spliceosome RNA helicase BAT1	0.77
6	P10645	CHGA	Chromogranin-A	0.73
7	P10909	CLU	Clusterin	0.63
8	P16870	CPE	Carboxypeptidase E	0.68
9	P01034	CST3	Cystatin-C	0.64
10	O43237	DYNC1LI2	Cytoplasmic dynein 1 light intermediate chain 2	0.78
11	P49327	FASN	Fatty acid synthase	1.32
12	P11413	G6PD	Glucose-6-phosphate 1-dehydrogenase	1.22
13	P02774	GC	Vitamin D-binding protein	0.63
14	P01275	GCG	Glucagon	0.61
15	Q06210	GFPT1	Glutamine—fructose-6-phosphate aminotransferase [isomerizing] 1	1.40
16	Q96IJ6	GMPPA	Mannose-1-phosphate guanyltransferase alpha	1.46
17	P21695	GPD1	Glycerol-3-phosphate dehydrogenase NAD+, cytoplasmic	1.21
18	Q9NZ01	GPSN2	Synaptic glycoprotein SC2	1.22
19	P49257	LMAN1	Protein ERGIC-53	1.25
20	Q96JB6	LOXL4	Lysyl oxidase homolog 4	0.75
21	O95299	NDUFA10	NADH dehydrogenase ubiquinone 1 alpha subcomplex subunit 10	1.22
22	P04181	OAT	Ornithine aminotransferase, mitochondrial	1.30
23	O95340	PAPSS2	Bifunctional 3'-phosphoadenosine 5'-phosphosulfate synthetase 2	1.37
24	Q9UHG2	PCSK1N	Proprotein convertase subtilisin/kexin type 1 inhibitor	0.64
25	P16519	PCSK2	Neuroendocrine convertase 2	0.69
26	Q63HM9	PLCXD3	Phosphatidylinositol-specific phospholipase C X domain-containing protein 3	1.25
27	Q13162	PRDX4	Peroxiredoxin-4	1.23
28	P28066	PSMA5	Proteasome subunit alpha type-5	1.41
29	Q16769	QPCT	Glutaminyl-peptide cyclotransferase	0.73
30	P02753	RBP4	Plasma retinol-binding protein	0.47
31	P05451	REG1A	Lithostathine-1-alpha	0.55
32	P13521	SCG2	Secretogranin II	0.73
33	Q8WXD2	SCG3	Secretogranin III	0.62
34	P05408	SCG5	Secretogranin V	0.56
35	O94855	SEC24D	Protein transport protein Sec24D	1.40
36	Q01105	SET	Protein SET	1.35
37	P17405	SMPD1	Acid sphingomyelinase	1.24
38	P02766	TTR	Transthyretin	0.55
39	Q13509	TUBB3	Tubulin beta-3 chain	1.56
40	Q8NBS9	TXNDC5	Thioredoxin domain-containing protein 5	1.31
41	O15240	VGF	Neurosecretory protein VGF	0.75

Differentially expressed proteins with FDR less than 0.15, and fold changes less than 0.8 or greater than 1.2

Of the proteins known to exist in or be closely associated with the immature secretory granule, almost all were detected and found to be significantly down-regulated in islets treated for 7 days with palmitate: CHGA (CGA), CPE, PCSK1N (ProSAAS), PCSK2 (PC2), SCG2 (SGII), SCG3 (SGIII), SCG5 (SGV, 7B2) and VGF. Two other well-known proteins associated with the secretory granule were detected and down-regulated in all six donors, but did not meet the test for adjusted significance: both PCSK1 (PC1/3) and CHGB (SCGI) had fold-changes of 0.74 and adjusted p-values equaling 0.16.

### Time-point comparisons for lipidomics: Differentially expressed lipids

**Fig 3:**
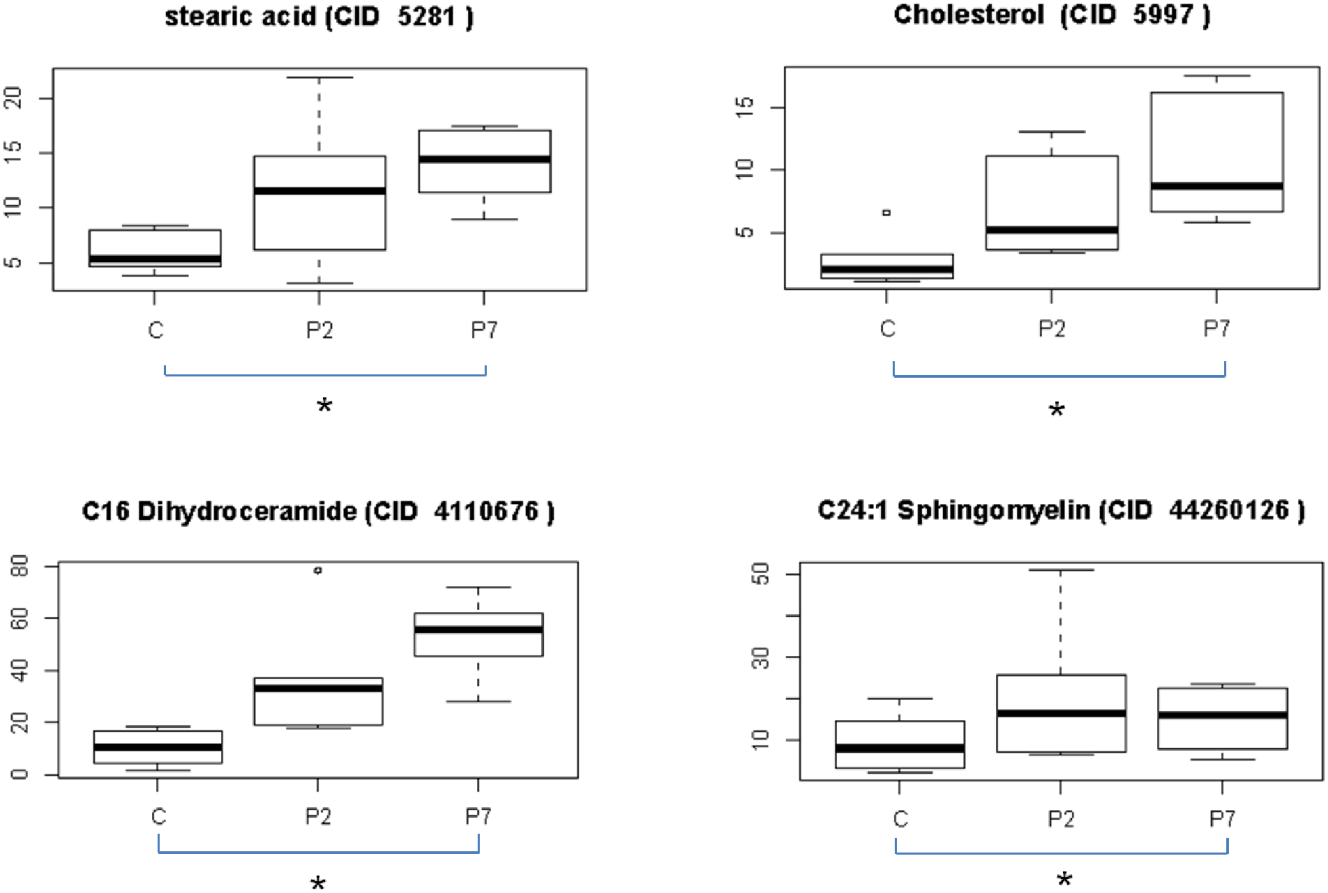
Differentially expressed islet lipids after palmitate treatment for 2 and 7 days. The PubChem Compound Identification (CID) is indicated in each case. Stearic acid and cholesterol were measured in μg, C16 dihydroceramide and C24:1 sphingomyelin were measured in pmol. *P-value of less than 0.05.

The same time-point comparisons were made for the lipidomic data as for the proteomics data. Many lipids were found to differ in concentration between different palmiatte exposure times using our stringent statistical approach, four were statistically significant. These were stearic acid, cholesterol, C16:0 dihydroceramide and C24:1 sphingomyelin, all of which occurred in the C vs. P7 comparison (Fig 3). All were substantially up-regulated at P7, when compared to control. The same four lipids normalized to islet number can be visualized in S1 Fig.

### Donor-by-donor pathway analysis based on proteomic data

**Fig 4:**
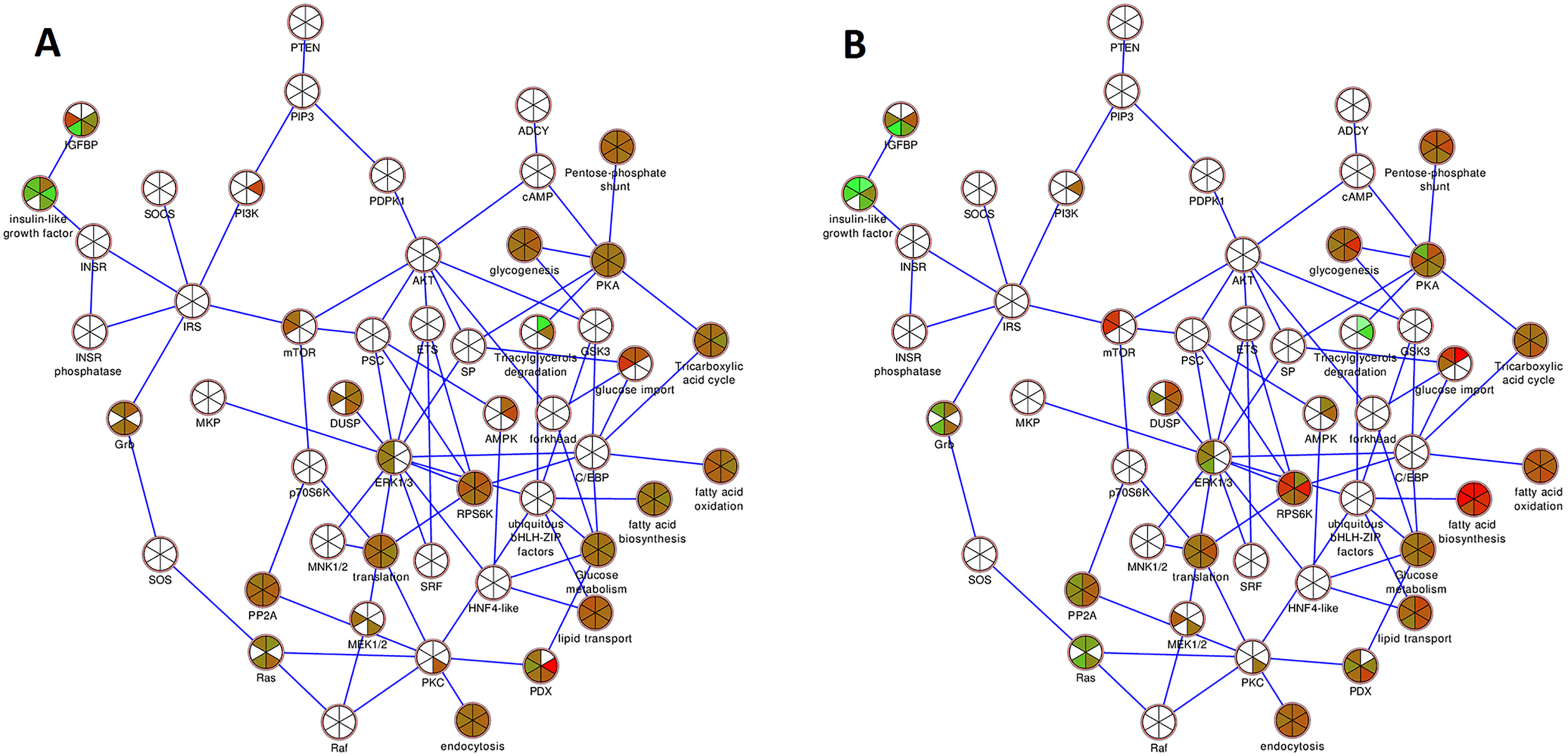
Insulin action, top scoring pathway for islet proteomics data. Down-regulated (green), up-regulated (red), unchanged (brown) and not detected (white) proteins when control (C) and P2 islets (A) and C and P7 islets (B) were compared. Each wedge in a node represents one of the six donors, the position of a particular donor’s wedge in a node is consistent throughout the figure.

**Fig 5:**
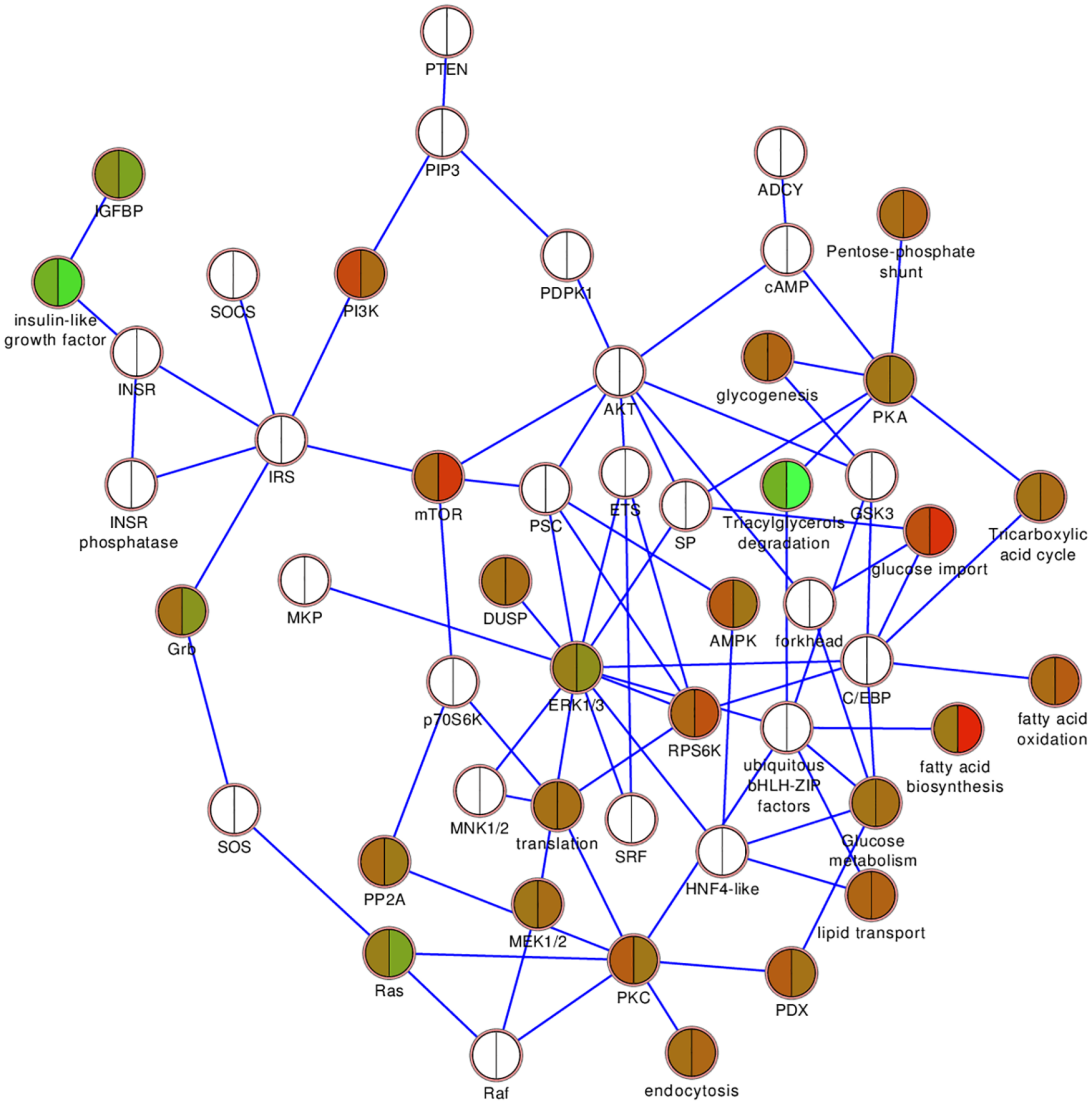
Insulin action pathway showing the averages over all six donors at P2 (left half of node) and P7 (right half of node). Down-regulated (green), up-regulated (red), unchanged (brown) and not detected (white) proteins are shown.

Proteins identified in the six donors ranged from 1278 to 1532 proteins. For each donor the corresponding protein data set was used in a pathway analysis. There was a high percentage of overlap of detected proteins with the insulin action pathway. This lead to significant p-values for the pathway in all six donors individually (Table 2). As a consequence the insulin action pathway was the top overall ranking pathway (Table 3). The insulin action pathway was then visualized with the donor expression data in Cytoscape (Figs 4 and 5).

**Table 2:** Enriched pathways based on the islet proteomics data set from a single donor.

Name	Total Entities	Expanded # of Entities	Overlap	Percent Overlap	p-value
Insulin Action	50	912	242	26	5.00E-47
EphrinR -> actin signaling	15	216	49	22	9.49E-18
Mitochondrial Protein Transport	25	111	45	40	1.82E-16
Actin Cytoskeleton Assembly	31	55	28	50	1.19E-13
B Cell Activation	62	847	159	18	6.74E-13
Tight Junction Assembly (Occludin)	38	294	69	23	9.14E-11
Ubiquitin-dependent Protein Degradation	15	125	38	30	1.26E-09
rRNA Transcription and Processing	35	292	58	19	1.96E-06
Notch Pathway	40	1487	214	14	4.38E-06
Adipocytokine Signaling	52	792	122	15	5.05E-05
Presentation of Endogenous Peptide Antigen	16	69	19	27	9.30E-05
Cell Cycle Regulation	135	2179	275	12	0.00346734
SomatostatinR -> ATF1/TP53 signaling	8	26	6	23	0.00731706
VasopressinR2 -> STAT signaling	7	7	3	42	0.00946125
FSHR -> CREB/ELK-SRF/GATA4 signaling	29	88	12	13	0.0159035
IGF1R -> STAT signaling	9	9	3	33	0.0204864
InsulinR -> STAT signaling	9	9	3	33	0.0204864
GlucagonR -> CREB/ELK-SRF/SP1 signaling	20	42	7	16	0.0233242
FSHR -> FOXO1A signaling	9	54	8	14	0.0302631

Enriched pathways identified using all proteins detected (n = 1429) in one of the donors. Proteins were uploaded into Pathway Studio and pathways with a p-value of less than 0.05 identified. “Total” refers to the total number of nodes in the pathway, some of which may be complex/container entities e.g. containing numerous proteins that are all of one functional class; “Expanded # of” refers to the total number of child concepts contained within the complex entities; “Overlap” refers to the number of proteins detected in the experimental dataset which overlap with the child concepts; “Percent” refers to the percentage of complex/container entities in which at least one experimental protein was detected.

**Table 3:** Pathway ranking scores for islet proteomics data.

Pathway	d1	d2	d3	d4	d5	d6	REP score donors	r1	r2	r3	r4	r5	r6	REP score random
Insulin Action	1	1	1	1	1	1	27.44	229	157	111	37	140	111	-1.01
EphrinR -> actin signaling	2	3	2	2	2	2	23.57	206	172	51	72	56	192	-0.52
Mitochondrial Protein Transport	3	2	6	3	3	4	21.37	225	116	27	91	88	87	0.52
B Cell Activation	5	4	3	5	4	3	20.86	37	149	191	80	200	130	-0.97
Actin Cytoskeleton Assembly	4	5	5	4	5	6	19.43	23	175	188	224	33	106	0.33
Ubiquitin-dependent Protein Degradation	7	6	4	6	7	5	18.91	213	56	149	232	209	135	-2.64
Tight Junction Assembly (Occludin)	6	7	7	7	6	7	17.86	56	219	239	163	178	22	-0.81
rRNA Transcription and Processing	8	8	9	8	8	9	16.81	38	160	56	112	126	98	0.56
Notch Pathway	9	10	8	9	9	8	16.58	59	210	153	136	67	14	1.23
Presentation of Endogenous Peptide Antigen	11	9	10	11	12	10	15.75	23	81	19	202	39	36	4.41
Adipocytokine Signaling	10	11	11	10	10	11	15.64	41	111	33	234	51	38	2.50
Cell Cycle Regulation	12	29	12	12	13	18	13.65	165	148	43	120	238	230	-2.11
AGER -> CREB/SP1 signaling	76	33	17	15	11	27	12.71	18	173	68	128	23	95	2.64
InsulinR -> STAT signaling	17	12	42	51	15	12	12.09	189	186	80	142	126	196	-2.47
ErythropoietinR -> STAT signaling	25	14	85	29	23	13	11.29	195	86	122	35	157	5	2.69
LeptinR -> STAT signaling	26	15	86	30	24	14	11.06	38	23	109	206	177	126	0.63
GlucagonR -> CREB/ELK-SRF/SP1 signaling	18	41	28	14	14	58	11.06	35	213	150	39	189	42	0.87

Pathway ranking scores for proteomics data obtained from six donors and six random ranks (r1 –r6). The overall score for the donors is the left REP score column (-log(e-value)), the score for the random ranks is the right REP score column (-log(e-value))

### Integrated lipidomics and proteomics pathways

**Fig 6:**
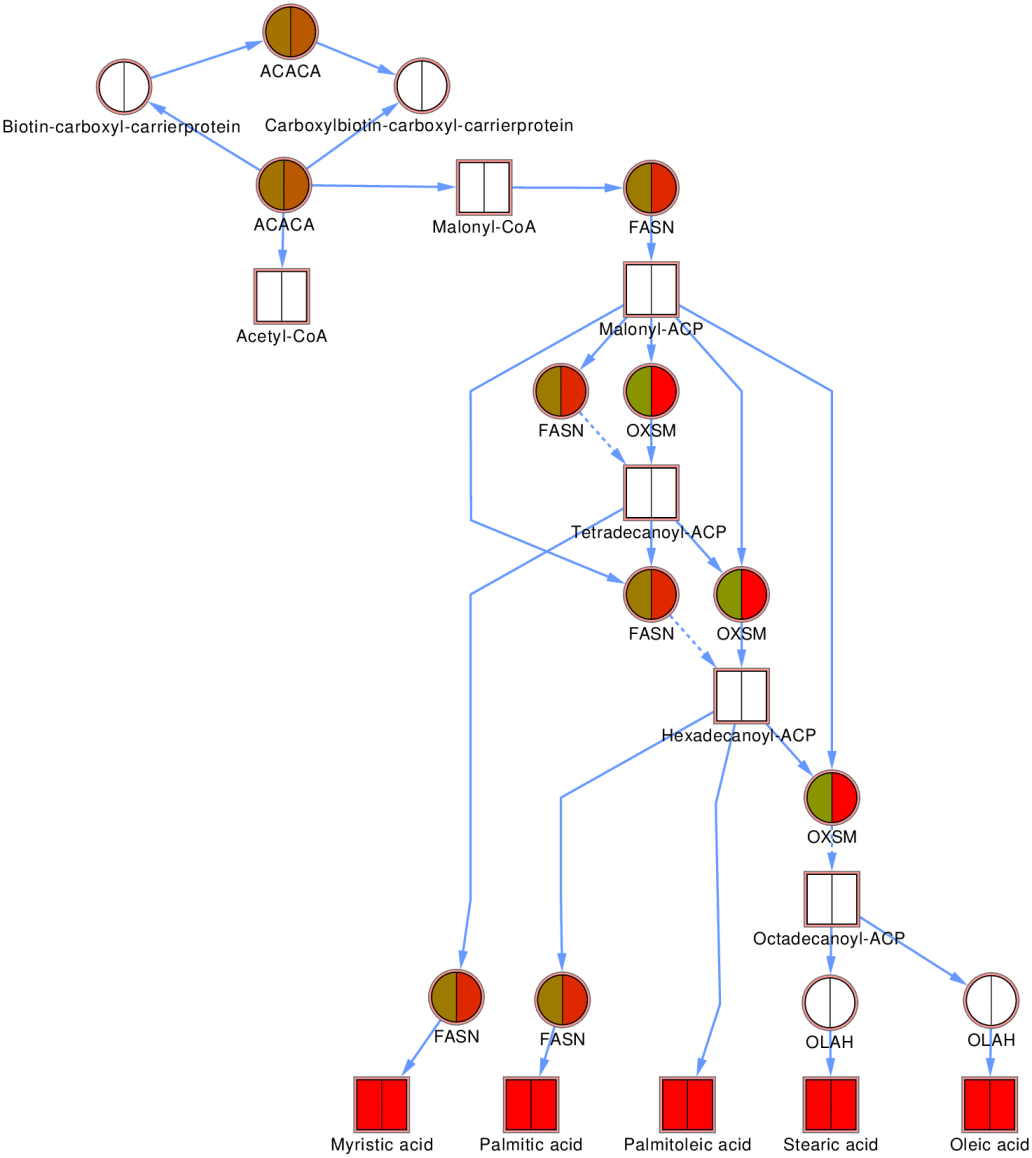
Fatty acid biosynthesis pathway. Proteins are indicated as circles, lipids as squares. The proteins in the pathway all catalyze multiple steps in the process and are thus shown multiple times. The averages over all six donors at P2 (left half of node) and P7 (right half of node) are shown. Down-regulated proteins/lipids are shown in green, up-regulated in red, unchanged in brown and not detected in white.

Fatty acid biosynthesis (Fig 6) was up-regulated as indicated by enhanced levels of most lipids at P2, which continued through to P7. Fatty acid synthase (FASN, detected in the present study) is a multi-enzyme protein, catalyzing multiple steps in the pathway. Mitochondrial 3-oxoacyl-ACP synthase (OXSM, detected), oleoyl-ACP hydrolase (OLAH, not detected) and acetyl-CoA carboxylase alpha (ACACA, detected), also catalyze multiple steps. FASN was not differentially expressed at P2, but up-regulated at P7, thus becoming up-regulated after the rise in lipids of the pathway.

**Fig 7:**
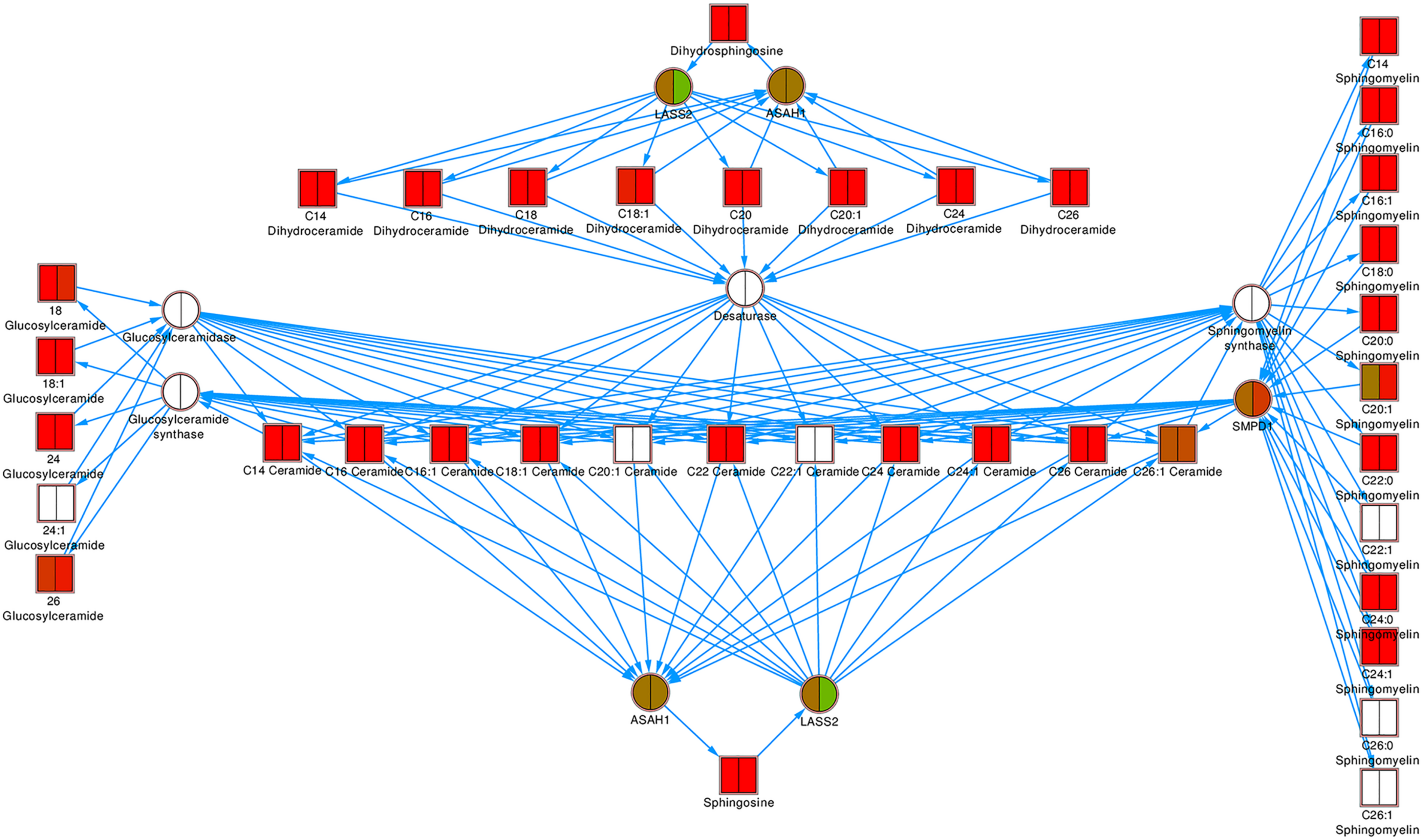
Sphingolipid metabolism pathway. Proteins are indicated as circles, lipids as squares. For the proteins, only one representative or no representative of each class was detected, and only this protein or no protein, respectively, was then shown. The averages over all six donors at P2 (left half of node) and P7 (right half of node) are represented. Down-regulated proteins/lipids are shown in green, up-regulated in red, unchanged in brown and not detected in white.

In sphingolipid metabolism, three of potentially 22 proteins were detected (Fig 7). These were ceramide synthase 2 (LASS2), acid ceramidase (ASAH1) and acid sphingomyelinase (SMPD1) and were used to represent other proteins in their respective classes in the figure. No glucosylceramidases, glucosylceramide synthases, desaturases, sphingomyelin synthases were detected. Almost all lipids were up-regulated, at both P2 and P7, although only two of these differences were statistically significant (Fig 7). Similarly to fatty acid biosynthesis, it takes longer for protein concentrations to change than those of lipids: at P7 LASS2 was down-regulated and SMPD1 was up-regulated. ASAH1 remained unchanged throughout.

## Discussion

In this study we have combined proteomic and lipidomic data sets obtained from human pancreatic islets. Whereas an untargeted approach was taken to generate proteomic data, a targeted one was employed for the lipidomic data. Combining proteomics and lipidomics is a developing field due to the significant knowledge gap that exists between these two fields: lipid subspecies are not well addressed in pathway databases and lipid databases do not easily give information on the proteins involved in lipid metabolism. In our study, we have combined this data for the first time for two relevant pathways in the beta-cell, namely fatty acid biosynthesis and sphingolipid metabolism pathways.

Glucose-stimulated insulin secretion from the islets increased on day 2 and decreased on day 7. At day 7 islet insulin content decreased and the proinsulin to insulin content ratio doubled. Amounts of cholesterol, stearic acid, C16 dihydroceramide and C24:1 sphingomyelin, obtained from the lipidomic screen, increased time-dependently in the palmitate-exposed islets. The proteomic screen identified matching changes in proteins involved in lipid biosynthesis indicating up-regulated cholesterol and lipid biosynthesis in the islets. Pathway analysis based on the protein and lipid expression profiles implicated autocrine effects of insulin as well as mitochondrial protein and cell cycle regulation in lipotoxicity.

Transcriptomics analysis has been applied on human islets exposed to palmitate [29]. We now extend the research area of human islets exposed to palmitate to gather protein data sets by the present proteomics approach. Further, in contrast to the transcriptomics study, the present study contains multiple time points and thus gives a thorough and dynamic picture of the cellular processes occurring within human pancreatic islets being treated with palmitate.

The findings show differentially expressed proteins after exposing isolated primary human islets to high palmitate levels, thus identifying proteins involved in the effect of saturated fatty acids. A potential drawback of using islets from human donors could be that epigenetic changes exists that make the findings more representative of the BMI, age or ethnic group that we studied. However, this kind of epigenetic effect has not been demonstrated and the fact that all cells are of high purity for islet preparations (85–95% of the cells are endocrine pancreas and not exocrine or other cell type) and they are exposed to the same conditions make the results of interest and more translatable to human physiology, while still keeping in mind that it is an *in-vitro* model. The alternative is a cell line with the same genetic background. We view it to as a strength, that there is genetic heterogeneity among the donors. With regards to age and BMI, all donors can be considered middle age and the BMIs within a similar range. It has also never been documented that there is a difference associated with starting the palmitate treatment 4 or 7 days after islet extraction from the donor.

The treatment of human islets with chronically elevated concentrations of palmitate under normoglycemic conditions has been shown to produce an up-regulation in steroid biosynthesis and thus palmitate-induced lipotoxicity [30]. However, while Malmgren et al [30] used a transcriptomics-based approach to detect the up-regulation of genes associated with steroid biosynthesis in clonal rodent beta-cells, our study validated those findings that palmitate treatment directly enhances cholesterol biosynthesis in human islet-cells. Specifically, we were able to identify significantly increased levels of cholesterol, as well as stearic acid, C16:0 dihydroceramide and C24:1 sphingomyelin. The lipid concentrations showed quite an inter-donor variation and therefore, having more donors would increase statistical power and likely increase the number of alterations in lipid species after 7 days of palmitate treatment. This is one of the drawback of the current approach when working with primary islets from human donors and not clonal cells, where biological variation will make it harder to identify differences and chances for repeating experiments on the same genetic background is lost. On the other hand the strength in this approach is that significant changes observed are much more likely to be translatable.

Palmitate-induced cholesterol accumulation is of interest as well as the induction of fatty acid elongation, which is evident in the increased levels of stearic acid measured in islets exposed to palmitate for 7 days. These events coincide with a decrease in insulin secretion when compared to the amplified secretion that was present at 2 days of palmitate exposure (Fig 1), as well as a significant decrease in cellular insulin content (Fig 2). A recent study showed that inhibiting fatty acid elongation and stearate formation highly affected the secretory capacity of beta-cells, where inhibiting elongation preserved insulin secretion [31]. This is consistent with the findings in our study, where increased fatty acid synthesis and elongation correlate with less secretion capabilities and less insulin content.

A further important finding was that many proteins containing membrane-binding domains, which target them to the lipid-rich microdomains of the trans-Golgi network (TGN) membrane and later sorting to immature secretory vesicles, were all significantly down-regulated in human islets treated with palmitate despite significantly elevated levels of cholesterol. These proteins include chromogranin A (CHGA), carboxypeptidase E (CPE), prohormone convertase 2 (PCSK2), secretogranin 2 (SCG2), secretogranin III (SCG3), and neurosecretory protein VGF, and proprotein convertase subtilisin/kexin type 1 inhibitor PCSK1N, which acts as a potent inhibitor of PCSK1, and secretogranin V (SCG5), which acts as a chaperone for PCSK2. Two other well-known proteins associated with the immature secretory granule were detected and down-regulated in all six donors, but did not meet the test for adjusted significance in our study: prohormone convertase 1/3 (PCSK1) and chromogranin B (CHGB). In experiments with ABCA1 –p/-p mice [32], cholesterol accumulation has been shown to lead to major changes in the Golgi region and insulin granule morphology. Interestingly, no changes in the SNARE proteins (SNAP-25, VAMP2, STX1A, and STX4) were detected, for which we also did not detect any changes in our experiments. These proteins form a fusion complex, which is responsible for second-phase insulin secretion, where the secretory granules fuse with the plasma membrane in response to increased intracellular Ca^2+^ concentrations [33]. These findings are of significance as they imply that the granule maturation process is affected, contributing to less newly synthesized granules. This in turn leads to less available active insulin stores to maintain blood glucose levels and therefore a diabetogenic situation [34, 35]. Additionally, in the clinical setting the increase in proinsulin/insulin ratio will further increase the demand of the already dysfunctional beta-cell. Thus, our model shows characteristics observed in obese pre-diabetic and diabetic individuals [36–38].

Our pathway-based analysis of the proteomics data supported these experimental findings, as insulin action was the pathway most enriched in differentially expressed proteins, which implicates autocrine effects of insulin signaling as a long-term effect of palmitate treatment and the observed lipid metabolism. The same has recently been reported in a cell model, where a mutation in the insulin receptor of beta-cells adverted the toxic effects induced by nutrition excess [39]. Other notable pathways affected include mitochondrial proteins turnover and ephrin and actin signaling. Alterations in lipid species are likely to affect mitochondrial function as well as the cell-to-cell contact and cytoskeleton fluidity. Ephrin ligands are interesting as they are bound to lipid rafts and alterations in the cellular lipid pool might affect ephrin signaling as well as membrane fluidity [40]. Ephrins are also postulated to be of importance in cell-to-cell communications, which are highly important in islet cells. Communication is of particular importance between the different cell types as postulated in research on islet-cells, where ephrins are crucial for beta-cell cross-talk and can even regulate the effects of glucose on glucagon secretion [41, 42]. Palmitate can reportedly also directly affect the actin cytoskeleton via downstream signaling of the membrane bound free fatty acid receptor 1 (FFAR1/GPR40) to protein kinase D1 [43].

A possible explanation for worsening in secretory capabilities is evident in the finding that insulin content is reduced by almost 50% in islets treated for 7 days with palmitate, as has been observed before in beta-cells given chronic palmitate treatment [4, 44, 45]. A potential explanation could be that islets are degranulated without compensatory mature granule formation and therefore also account for the concomitant decrease in granular proteins. Ishikawa et al. attempted to determine the role of cholesterol synthesis in pancreatic beta-cells by overexpressing the sterol-regulatory element binding protein 2 (SREBP-2) in mice. These mice became diabetic with inadequate insulin secretion with islets that had very high levels of intra-islet cholesterol and significantly reduced insulin content [46]. The same phenomena were found in our study. While degranulation or reduced insulin content most likely contributes to decreased insulin secretion it does not rule out a role for cholesterol and lipid accumulation in decreasing secretory capabilities given the above mentioned importance of cholesterol in secretion. In a study on the INS-1 832/13 beta-cell line it was found that high glucose induced a decrease in insulin content with concomitant decrease in secretory capabilities. However, when the cells were maintained at normal glucose levels, the secretory function was regained despite insulin content remaining low [47]. Furthermore, exposure to high levels of glucose increased cholesterol biosynthesis in rat islets [48], and high glucose together with high palmitate increased cholesterol formation in the rat beta-cell line INS 832/13 [49]. Therefore, it is possible that this accumulation of cholesterol in palmitate-treated islets affects secretion capabilities both directly by depleting granular stores and indirectly by affecting granular formation. Further research would be needed to dissect the roles of the different lipid species affected in this study in granular formation and function.

## Conclusions

The current study, where results were obtained by combining omics data, demonstrates that elevated palmitate levels in the presence of fasting glucose concentrations promote increased intracellular cholesterol formation in human pancreatic islets. This is of importance given the aforementioned role of cholesterol and lipid species in secretory granule formation and function. Taken together our study presents a novel way of combining lipid- and proteomics data to map metabolic and functional changes. Our findings indicate that chronic palmitate treatment elevates intracellular cholesterol and lipid species and affects autocrine insulin signaling. These changes are associated with a decrease in insulin granule amount or maturation as well as an initial rise followed by subsequent decline in insulin secretion in response to glucose in isolated human islets. Thereby a plausible mechanism for how chronic saturated free fatty acids could affect beta-cells during obesity and lead to the development of T2DM is presented. We think that our attempt to combine proteomics and lipidomics analysis on primary cells was in large parts successful, especially considering the challenge in combining such data. Still much further technological software and curation development is needed in the field to allow for better integration of protein and lipidomics datasets in general. We conclude that the current study demonstrates that the combination of the two omics approaches can give valuable and broad information on biological pathways in disease models.

## Supporting information

S1 Fig Differentially expressed islet lipids normalized to islet number. Cells remained untreated in control conditions, or were treated with palmitate for 2 or 7 days. Normalization was performed to 100 islets in each case.
(TIFF)
