# Supplementary figures and images for: Combined lipidomic and proteomic analysis of isolated human islets exposed to palmitate reveals time-dependent changes in insulin secretion and lipid metabolism
Source: PLoS One. 2017 Apr 27;12(4):e0176391. doi: 10.1371/journal.pone.0176391 (PMC5407795; doi:10.1371/journal.pone.0176391)

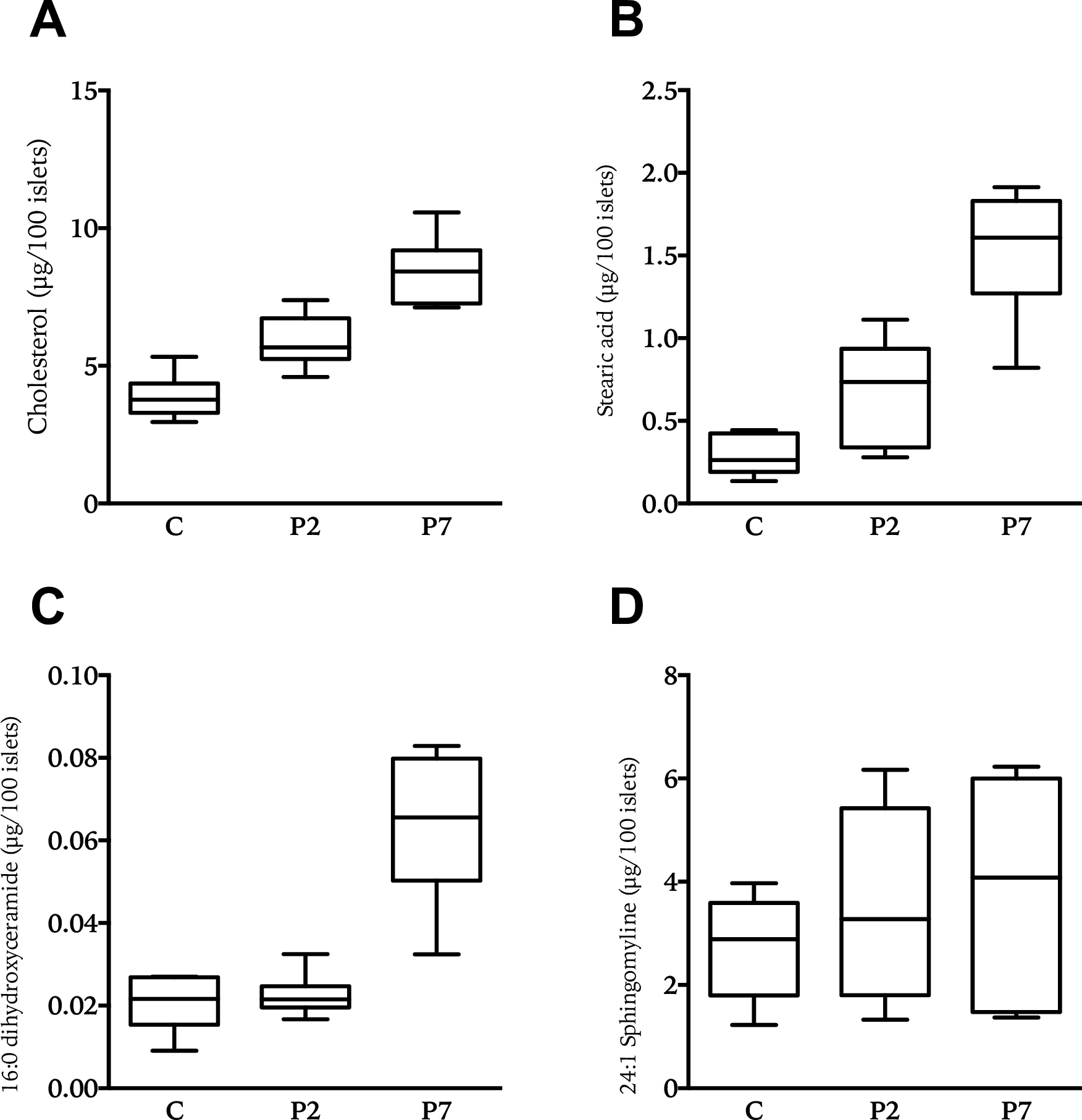

Supplement: S1 Fig — Cells remained untreated in control conditions, or were treated with palmitate for 2 or 7 days. Normalization was performed to 100 islets in each case. (TIFF) [file pone.0176391.s001.tiff]
